# Supplementary material for: A trait-based root acquisition-defence-decomposition framework in angiosperm tree species
Source: Nat Commun. 2024 Jun 21;15:5311. doi: 10.1038/s41467-024-49666-3 (PMC11192760; doi:10.1038/s41467-024-49666-3)
Supplement: Supplementary file 1 — Supplementary Information [file 41467_2024_49666_MOESM1_ESM.pdf]

Supplementary information for

A trait-based root acquisition-defence-decomposition framework in angiosperm tree species

Jiajia Zheng[1,2,3], Grégoire T. Freschet[4], Leho Tedersoo[5,6], Shenggong Li[1,3], Han Yan[7,8], Lei Jiang[9], Huimin Wang[1,2,3], Ning Ma[1,3], Xiaoqin Dai[1,2], Xiaoli Fu[1,2,3] & Liang Kou[1,2,3]*

Corresponding author: Liang Kou

Email: koul@igsnrr.ac.cn

Article type: Primary research articles

This file includes:

Supplementary Figures 1-8
Supplementary Tables 1-6

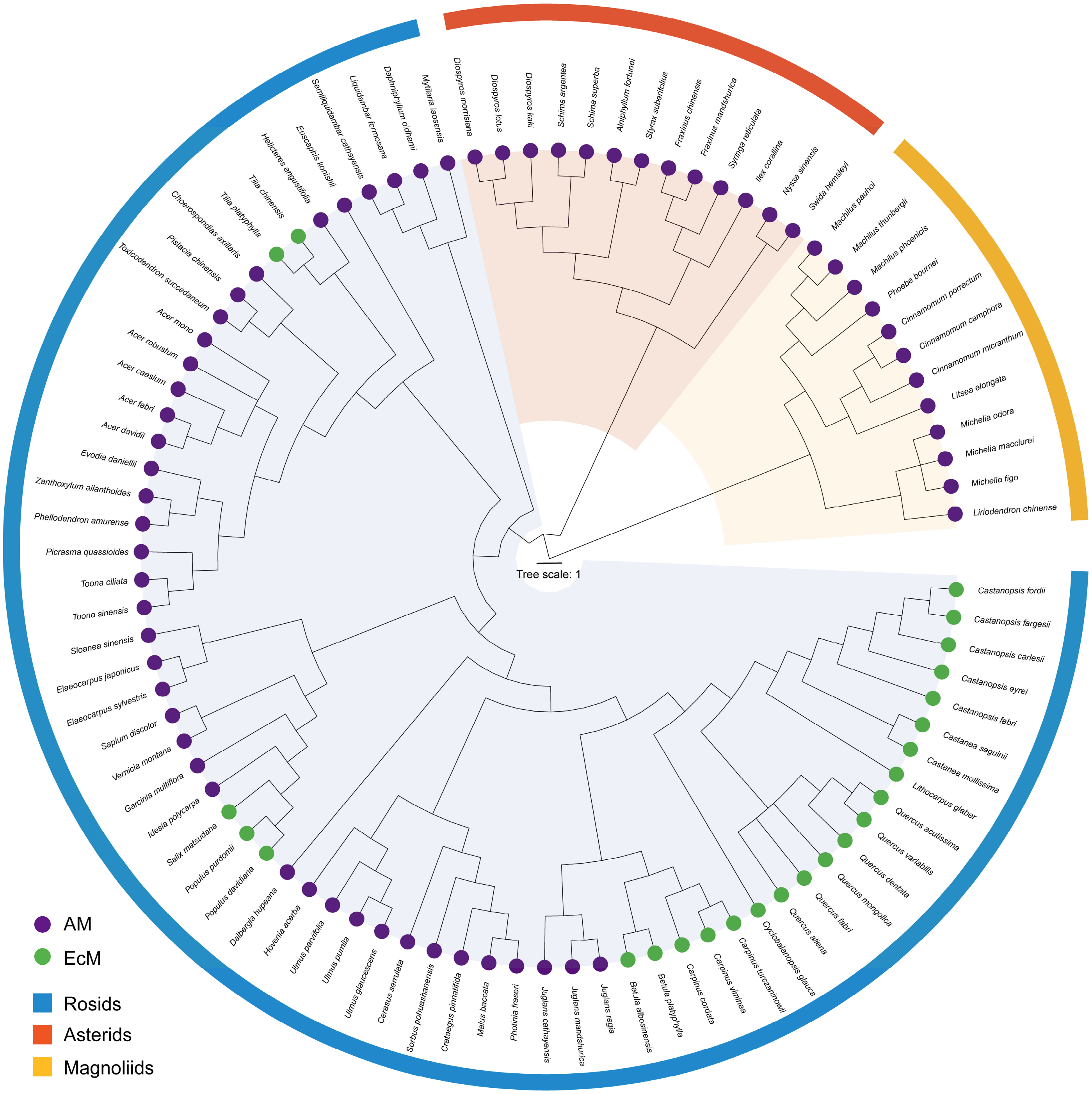

Supplementary Fig. 1 | Phylogenetic tree of 90 tree species used in this study. The outer circle colors for the Latin names represent different clades, and the solid dots at the tip of the phylogenetic branch represents the mycorrhizal type, purple for arbuscular mycorrhizal (AM) tree species, and green for ectomycorrhizal (EcM) tree species. Specific information about the clade and mycorrhizal type for each species can be found in Supplementary Data 1.

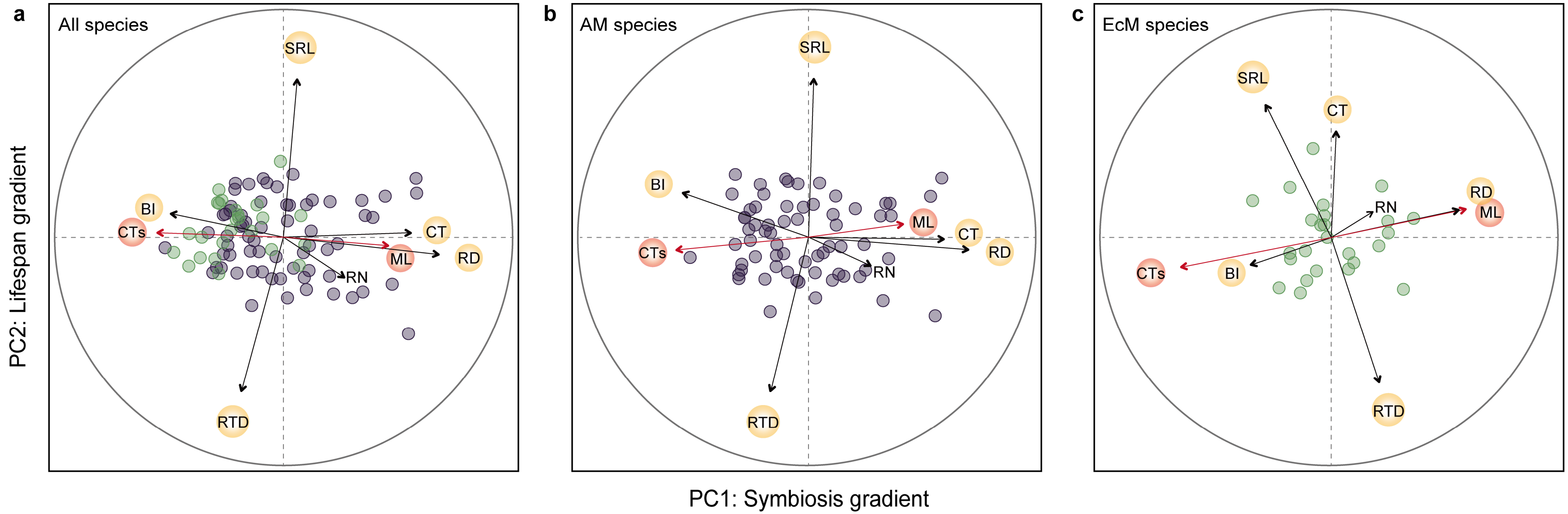

Supplementary Fig. 2 | The test of the root economic space (RES) using the eight traits (RD, SRL, RTD, RN, BI, CT, CTs, ML). Root trait biplots of phylogenetic principal component analysis (pPCA) for (**a**) all 90 tree species; (**b**) 65 arbuscular mycorrhizal (AM) species (purple circle); (**c**) 25 ectomycorrhizal (EcM) species (green circle). For more details on pPCA scores, see Supplementary Table 5. Both CTs and ML are located on the 'symbiosis gradient'(PC1). RD, root diameter; SRL, specific root length; RTD, root tissue density; RN, root nitrogen concentration; BI, branching intensity; CT, cortex thickness; CTs, condensed tannins concentration; ML, mass loss.

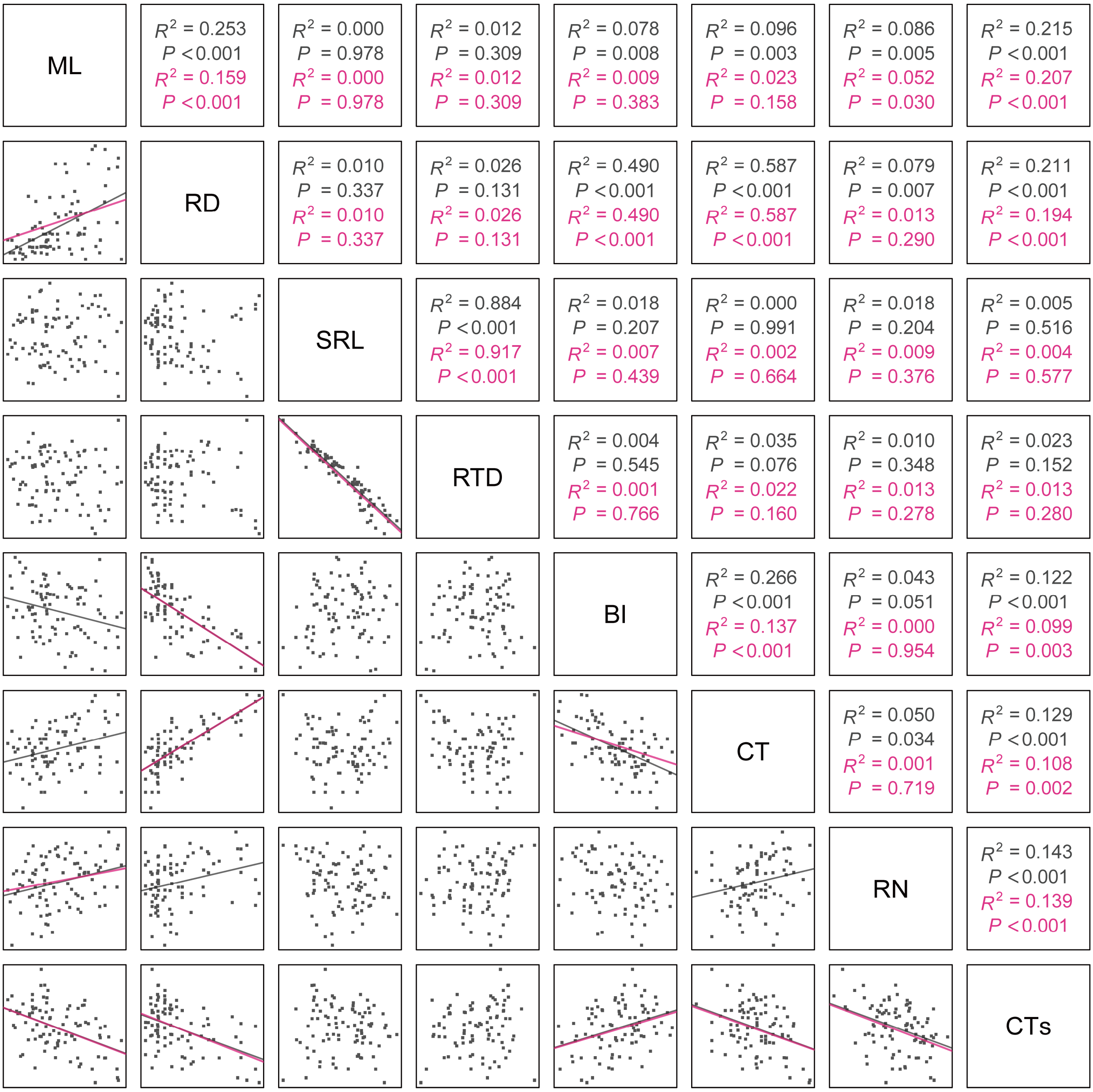

Supplementary Fig. 3 | Pairwise correlations among root traits for all tree species. The bivariate relationships among root traits for all tree species (*n* = 90) are assessed using ordinary least squares (OLS) models and phylogenetic generalized least squares (PGLS) models. Significant correlations are represented by regression lines coloured in grey for OLS models and in red for PGLS models (lower triangle). Correlation coefficients are presented in the corresponding colours for the two regression models (upper triangle). ML, mass loss; RD, root diameter; SRL, specific root length; RTD, root tissue density; BI, branching intensity; CT, cortex thickness; RN, root nitrogen concentration; CTs, condensed tannins concentration. Statistical significance in the regression model was determined using two-sided *t*-tests.

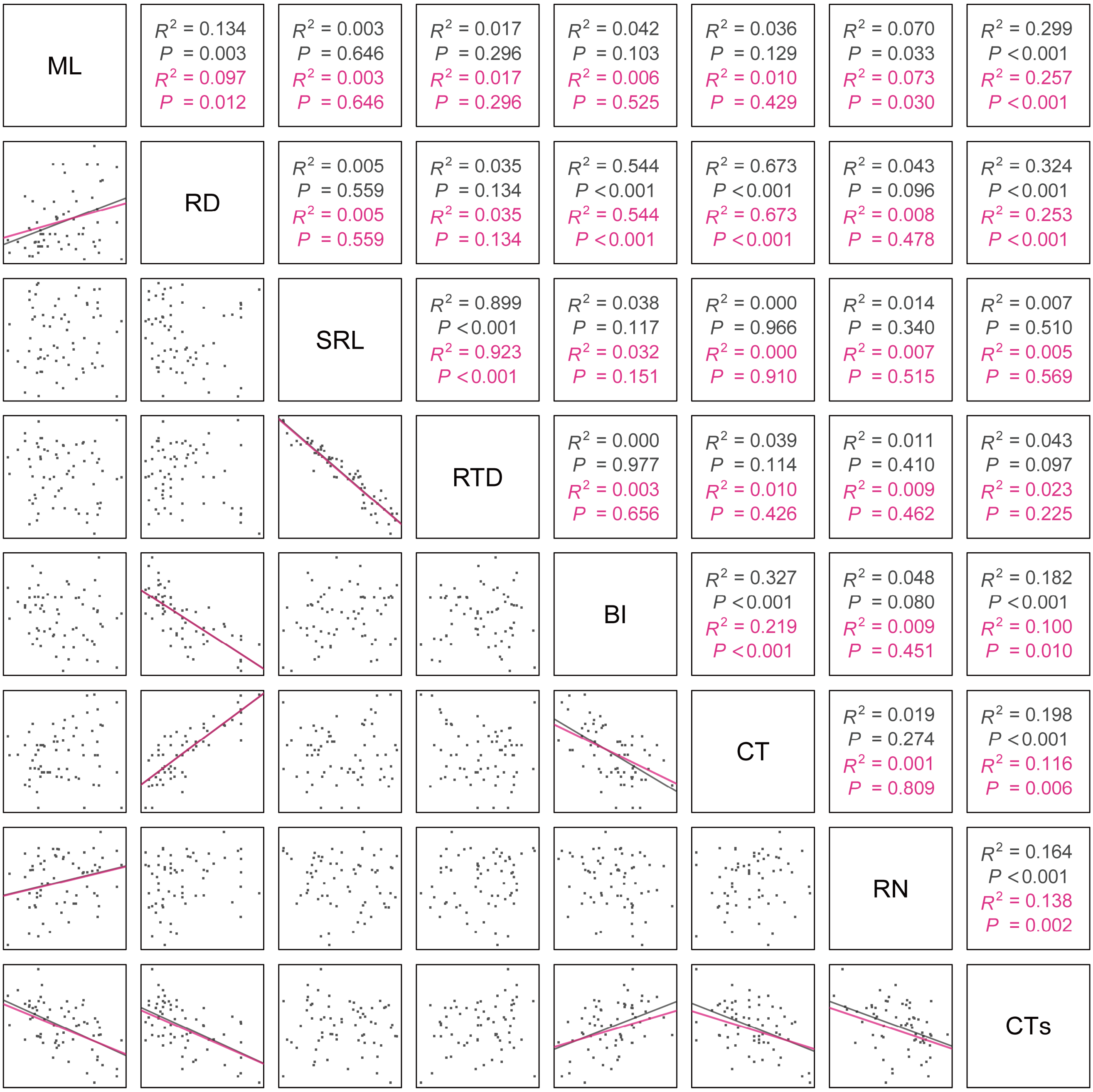

Supplementary Fig. 4 | Pairwise correlations among root traits for arbuscular mycorrhizal tree species. The bivariate relationships among root traits for arbuscular mycorrhizal tree species (n = 65) are assessed using ordinary least squares (OLS) models and phylogenetic generalized least squares (PGLS) models. Significant correlations are represented by regression lines coloured in grey for OLS models and in red for PGLS models (lower triangle). Correlation coefficients are presented in the corresponding colours for the two regression models (upper triangle). ML, mass loss; RD, root diameter; SRL, specific root length; RTD, root tissue density; BI, branching intensity; CT, cortex thickness; RN, root nitrogen concentration; CTs, condensed tannins concentration. Statistical significance in the regression model was determined using two-sided t-tests.

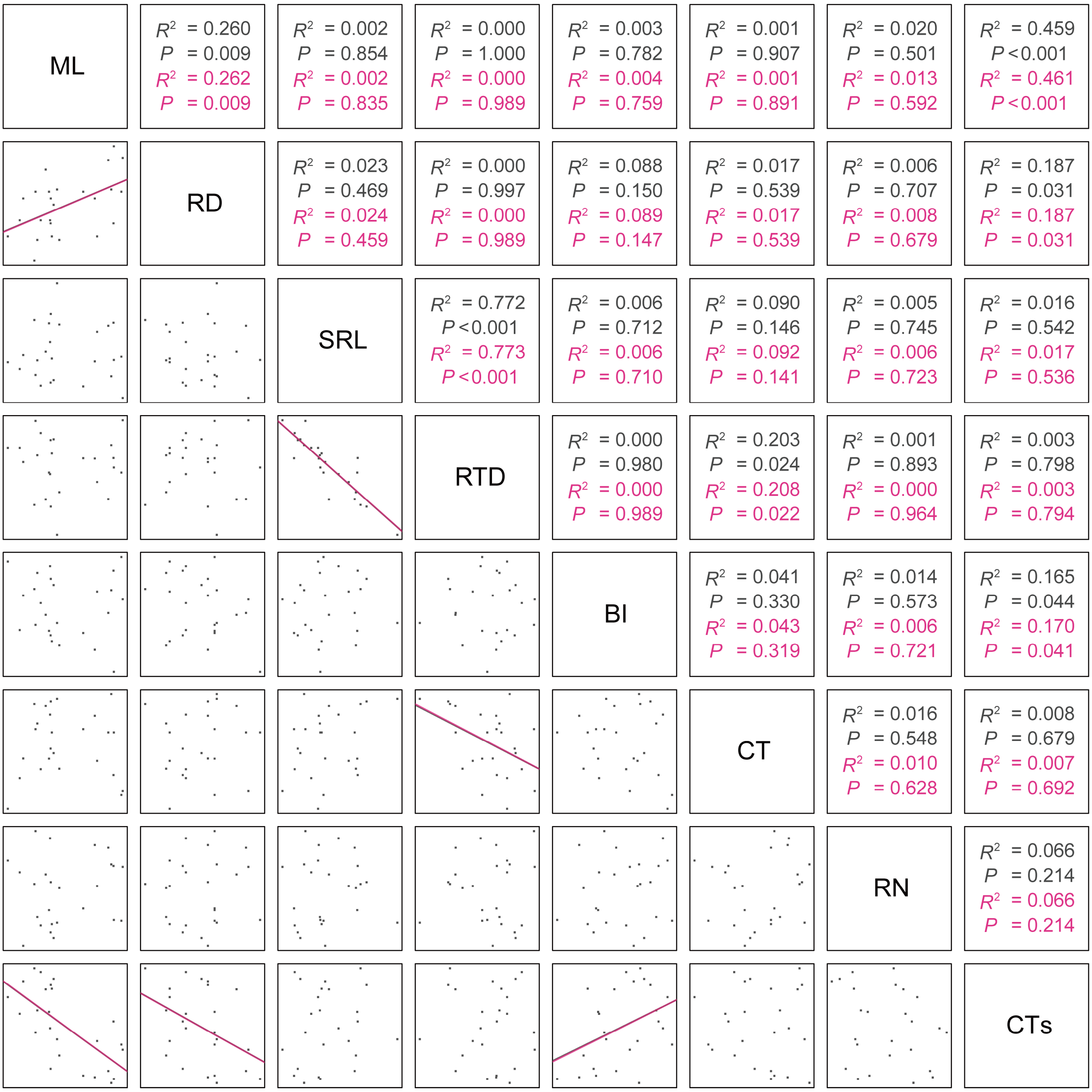

Supplementary Fig. 5 | Pairwise correlation among root traits for ectomycorrhizal tree species. The bivariate relationships among root traits for ectomycorrhizal tree species (*n* = 25) are assessed using ordinary least squares (OLS) models and phylogenetic generalized least squares (PGLS) models. Significant correlations are represented by regression lines coloured in grey for OLS models and in red for PGLS models (lower triangle). Correlation coefficients are presented in the corresponding colours for the two regression models (upper triangle). ML, mass loss; RD, root diameter; SRL, specific root length; RTD, root tissue density; BI, branching intensity; CT, cortex thickness; RN, root nitrogen concentration; CTs, condensed tannins concentration. Statistical significance in the regression model was determined using two-sided *t*-tests.

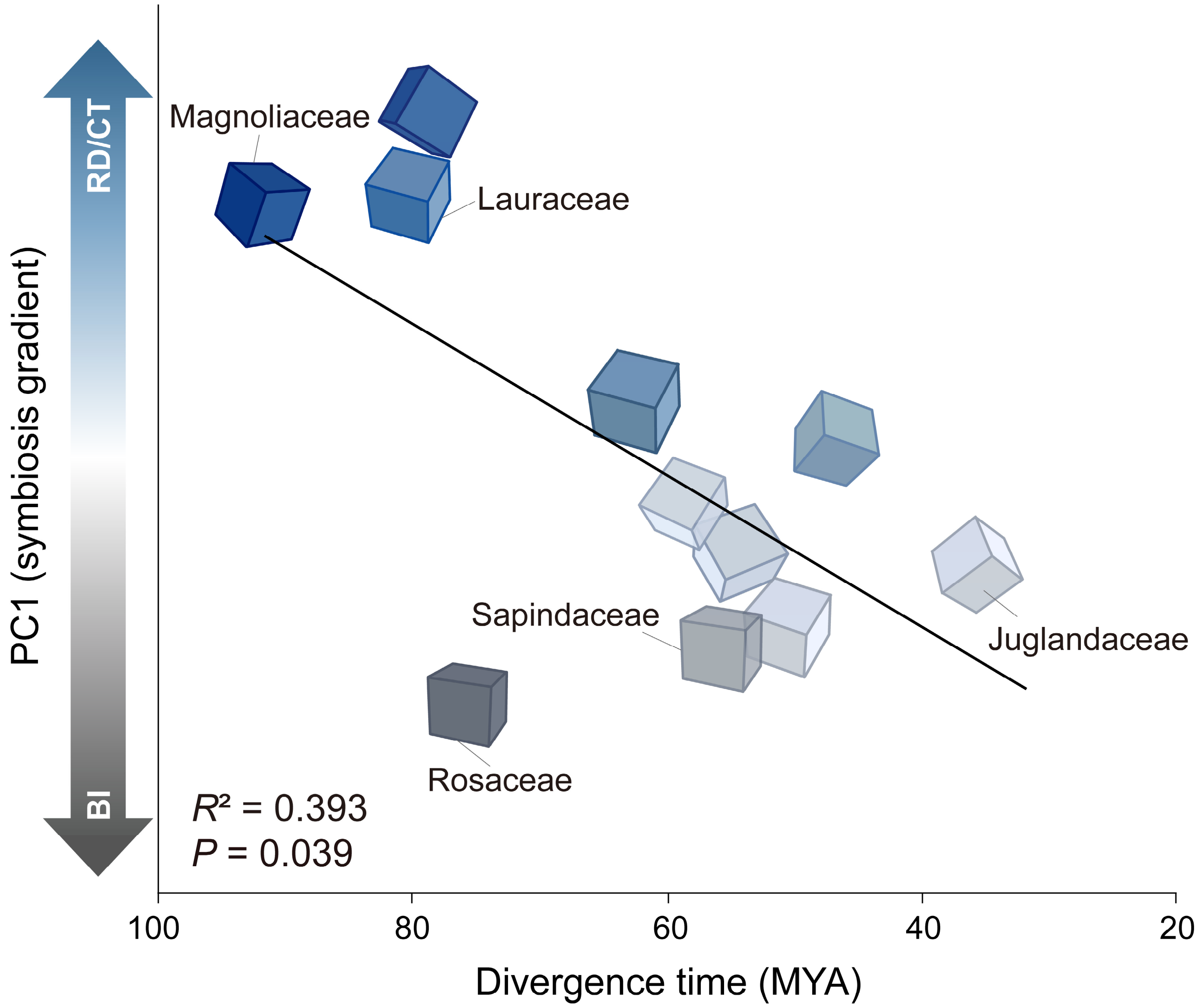

Supplementary Fig. 6 | Relationships between the scores of 'symbiosis gradient' (the first principal component, PC1) and the divergence time in evolutionary history (MYA, million years ago), of major AM-taxonomic groups (*n* = 11) at the family level (only families with at least three species are retained). The cubes represent a multidimensional strategy along the 'acquisition-defence-decomposition' continuum driven by the 'symbiosis gradient'. Solid lines indicate regression lines (*F*-test). AM, arbuscular mycorrhizal. Notes: The families involved here refer to those to which the AM species in this study belong, and does not mean that all species in this family are AM.

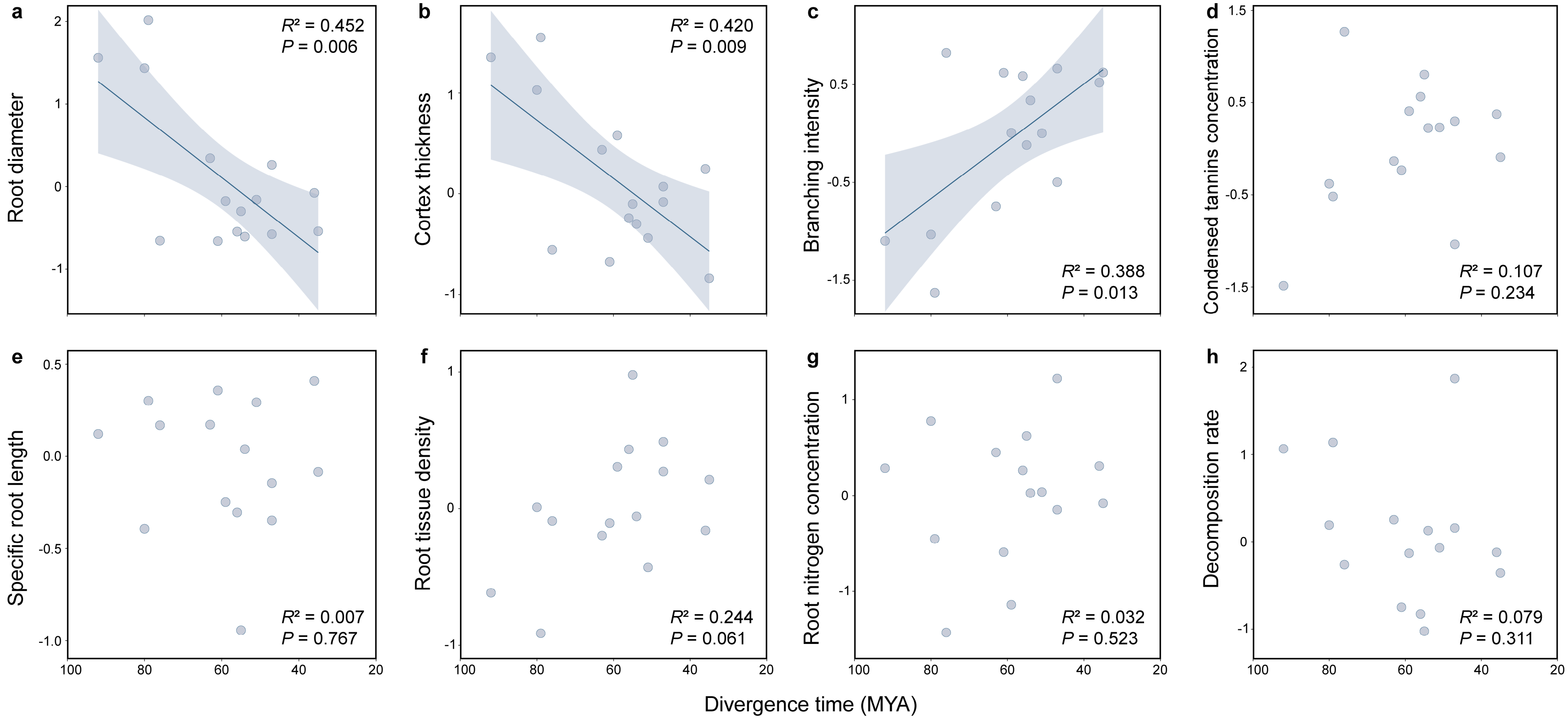

Supplementary Fig. 7 | Relationships between root traits and divergence time (MYA, million years ago) of major taxonomic groups (*n* = 15) at the family level (only families with at least three species were retained). Only root diameter (**a**), cortex thickness (**b**), and branching intensity (**c**) of absorptive roots showed significant evolution trend, which are represented by regression lines with the shaded areas indicating 95% confidence intervals (*F*-test). Regression lines were not shown for root traits with insignificant evolutionary trend (**d-h**).

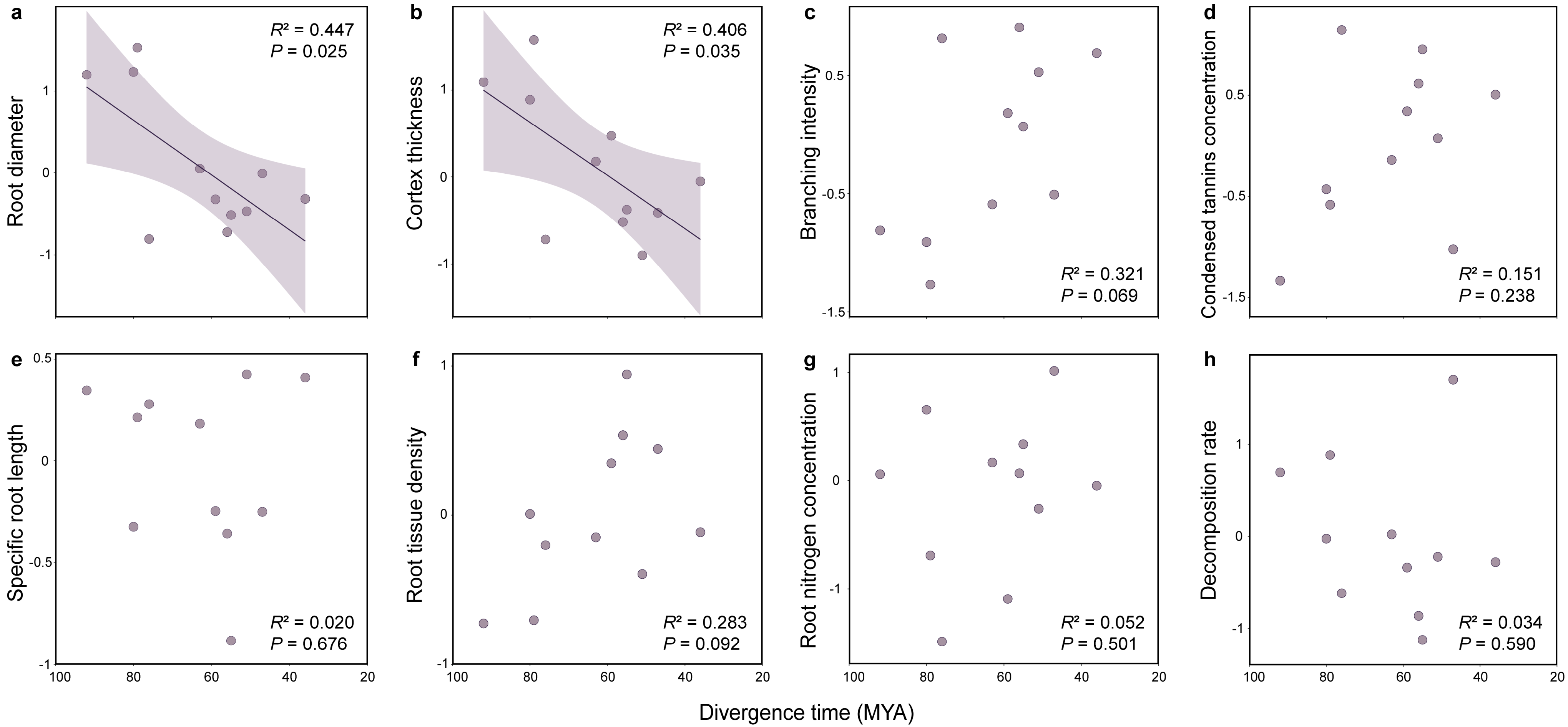

Supplementary Fig. 8 | Relationships between root traits and and divergence time (MYA, million years ago) of major arbuscular mycorrhizal-taxonomic groups (*n* = 11) at the family level (only families with at least three species were retained). Only root diameter (**a**) and cortex thickness (**b**) of absorptive roots showed significant evolution trend, which are represented by regression lines with the shaded areas indicating 95% confidence intervals (*F*-test). Regression lines were not shown for root traits with insignificant evolutionary trend (**c-h**).

Supplementary Table 1 | Variations and phylogenetic signals (Blomberg's *K*) of absorptive root traits in angiosperm tree species.

Root trait	Units	All angiosperm tree species (*n* = 90)						Tree species (*n* = 70) from the major families	
		Min	Max	Mean	CV%	*K*-value	*P*-value	*K*-value	*P*-value
RD	mm	0.320	0.600	0.376	16.0	**0.088**	0.036	**0.086**	0.025
SRL	m g^{-1}	9.600	215.780	69.118	72.7	0.018	0.411	0.015	0.415
RTD	g cm^{-3}	0.030	0.740	0.240	72.8	0.024	0.318	0.020	0.320
BI	branches cm^{-1}	0.770	13.299	4.594	65.4	0.045	0.111	0.039	0.089
CT	mm	0.040	0.365	0.185	37.6	0.068	0.071	**0.066**	0.036
RN	%	0.990	2.630	1.852	22.6	**0.101**	0.008	**0.081**	0.007
CTs	%	0.970	8.350	3.392	43.9	**0.120**	0.005	**0.100**	0.004
ML	%	6.830	35.860	16.656	37.0	0.051	0.109	0.043	0.097

RD, root diameter; SRL, specific root length; RTD, root tissue density; BI, branching intensity; CT, cortex thickness; RN, root nitrogen concentration; CTs, condensed tannins concentration; ML, mass loss. Min, minimum value; Max, maximum value; CV, coefficient of variation; The reported *P* values result from the randomization test (999 simulations), and significant *K* values are in bold ($P < 0.05$).

Supplementary Table 2 | Variations of absorptive root traits in 65 arbuscular mycorrhizal (AM) tree species and 25 ectomycorrhizal (EcM) tree species.

Traits	Units	Mycorrhizal type	Mean (SE)	Statistic	CV (%)
RD	mm	AM	0.390[a] (0.008)	$F_{1,88}$ = 16.180	16.7
		EcM	0.340[b] (0.003)	***P* < 0.001**	4.7
SRL	m g^{-1}	AM	68.084 (6.552)	$F_{1,88}$ = 0.662	77.6
		EcM	71.807 (8.735)	*P* = 0.418	60.8
RTD	g cm^{-3}	AM	0.242 (0.023)	$F_{1,88}$ = 0.056	76.9
		EcM	0.235 (0.029)	*P* = 0.813	61.8
BI	branches cm^{-1}	AM	3.682[b] (0.298)	$F_{1,88}$ = 13.680	65.2
		EcM	6.963[a] (0.633)	***P* < 0.001**	45.5
CT	mm	AM	0.199[a] (0.008)	$F_{1,88}$ = 12.500	32.1
		EcM	0.147[b] (0.014)	***P* < 0.001**	47.8
RN	%	AM	1.938[a] (0.053)	$F_{1,88}$ = 6.963	22.2
		EcM	1.629[b] (0.057)	***P* = 0.009**	17.6
CTs	%	AM	3.504 (0.193)	$F_{1,88}$ = 0.215	44.5
		EcM	3.100 (0.253)	*P* = 0.644	40.9
ML	%	AM	17.858[a] (0.810)	$F_{1,88}$ = 12.350	36.6
		EcM	13.532[b] (0.714)	***P* < 0.001**	26.4

RD, root diameter; SRL, specific root length; RTD, root tissue density; BI, branching intensity; CT, cortex thickness; RN, root nitrogen concentration; CTs, condensed tannins concentration; ML, mass loss. Significant differences between means were compared using ANOVA (two-sided statistical test) based on Z-scores of the traits ($P < 0.05$). Different letters indicate significant differences between the two mycorrhizal types.

Supplementary Table 3 | Results of phylogenetic principal component analyses based on 6 root traits for all species, arbuscular mycorrhizal (AM) species, and ectomycorrhizal (EcM) species. Displayed are the Eigenvalue and the proportion of variation explained by each principal component (PC) and the loadings of the root traits.

		**PC1**	**PC2**	**PC3**	**PC4**
**All species**	Eigenvalue	2.175	1.977	0.956	0.616
n = 90	Variance	0.363	0.329	0.159	0.103
	RD	0.891	-0.232	-0.040	0.083
	SRL	0.184	0.967	0.125	-0.057
	RTD	-0.364	-0.919	-0.071	0.062
	RN	0.168	-0.263	0.945	-0.089
	BI	-0.714	0.258	0.196	0.598
	CT	0.823	-0.095	-0.038	0.487
**AM species**	Eigenvalue	2.348	2.006	0.957	0.494
n = 65	Variance	0.391	0.334	0.160	0.082
	RD	0.938	0.095	-0.070	0.072
	SRL	-0.146	0.974	0.121	-0.063
	RTD	-0.054	-0.988	-0.073	0.070
	RN	0.222	-0.177	0.958	0.049
	BI	-0.817	0.118	0.039	0.546
	CT	0.853	0.159	-0.119	0.425
**EcM species**	Eigenvalue	2.136	1.417	1.073	0.701
n = 25	Variance	0.356	0.236	0.179	0.117
	RD	0.033	0.767	0.190	0.593
	SRL	-0.908	-0.234	-0.055	0.160
	RTD	0.950	0.047	0.124	-0.178
	RN	-0.095	0.010	0.951	-0.111
	BI	0.036	-0.781	0.301	0.298
	CT	-0.629	0.402	0.151	-0.436

RD, root diameter; SRL, specific root length; RTD, root tissue density; RN, root nitrogen concentration; BI, branching intensity; CT, cortex thickness.

Supplementary Table 4 | One-way ANOVA (two-sided statistical test) used to test the effect of mycorrhizal type on the score of 'symbiosis gradient' and 'lifespan gradient' of 90 tree species.

Term	Mycorrhizal type		
	F	*df*	*P*
'symbiosis gradient' (PC1)	18.180	1, 88	< 0.001
'lifespan gradient' (PC2)	3.507	1, 88	0.064

Arbuscular mycorrhizal species (n = 65), Ectomycorrhizal species (n = 25).

Supplementary Table 5 | Results of phylogenetic principal component analyses based on 8 root traits for all species, arbuscular mycorrhizal (AM) species, and ectomycorrhizal (EcM) species. Displayed are the Eigenvalue and the proportion of variation explained by each principal component (PC) and the loadings of the root traits.

		**PC1**	**PC2**	**PC3**	**PC4**
**All species**	Eigenvalue	2.722	1.983	1.208	0.742
n = 90	Variance	0.340	0.248	0.151	0.093
	RD	0.873	-0.107	-0.274	0.080
	SRL	0.078	0.981	0.113	-0.071
	RTD	-0.237	-0.960	-0.012	0.042
	RN	0.344	-0.251	0.681	-0.515
	BI	-0.637	0.146	0.426	0.176
	CT	0.719	0.028	-0.372	-0.147
	CTs	-0.711	0.027	-0.394	0.052
	ML	0.592	-0.053	0.425	0.639
**AM species**	Eigenvalue	2.907	2.022	1.278	0.717
n = 65	Variance	0.340	0.248	0.151	0.093
	RD	0.900	0.095	0.288	0.056
	SRL	0.030	0.986	-0.008	-0.111
	RTD	-0.213	-0.962	-0.080	0.080
	RN	0.351	-0.178	-0.676	-0.588
	BI	-0.709	0.274	-0.313	0.131
	CT	0.764	-0.013	0.423	-0.086
	CTs	-0.746	-0.079	0.386	-0.043
	ML	0.533	0.081	-0.554	0.568
**EcM species**	Eigenvalue	2.339	2.123	1.171	0.924
n = 25	Variance	0.292	0.265	0.146	0.116
	RD	0.730	0.174	0.109	0.137
	SRL	-0.374	0.836	-0.137	0.209
	RTD	0.273	-0.909	-0.014	-0.209
	RN	0.238	0.162	-0.702	-0.596
	BI	-0.460	-0.173	-0.712	0.257
	CT	0.029	0.667	0.212	-0.478
	CTs	-0.858	-0.187	0.073	0.008
	ML	0.767	0.177	-0.301	0.411

RD, root diameter; SRL, specific root length; RTD, root tissue density; RN, root nitrogen concentration; BI, branching intensity; CT, cortex thickness; CTs, condensed tannins concentration; ML, mass loss.

Supplementary Table 6 | Results of the ordinary least squares regression among the scores of 'lifespan gradient', condensed tannins concentration and decomposition rate (*F*-test).

	All species (*n* = 90)		AM species (*n* = 65)		EcM sepcies (*n* = 25)	
	R^2	*P*	R^2	*P*	R^2	*P*
SRL-RTD axis scores-CTs	0.008	0.417	0.022	0.244	0.002	0.846
SRL-RTD axis scores-decomposition rate	0.015	0.253	0.008	0.469	0.001	0.908

AM, arbuscular mycorrhizal; EcM, ectomycorrhizal; CTs, condensed tannins concentration.
