# Supplementary material for: A trait-based root acquisition-defence-decomposition framework in angiosperm tree species
Source: Nat Commun. 2024 Jun 21;15:5311. doi: 10.1038/s41467-024-49666-3 (PMC11192760; doi:10.1038/s41467-024-49666-3)
Supplement: Supplementary file 3 — Description of Additional Supplementary Files [file 41467_2024_49666_MOESM3_ESM.pdf]

Description of Additional Supplementary Files

File name: Supplementary Data 1

Description:

Information on 90 angiosperm tree species used in this study, including site, mycorrhizal type, taxonomic information (species, genus, family, order, superorder), and forested biome.
