# Supplementary material for: A trait-based root acquisition-defence-decomposition framework in angiosperm tree species
Source: Nat Commun. 2024 Jun 21;15:5311. doi: 10.1038/s41467-024-49666-3 (PMC11192760; doi:10.1038/s41467-024-49666-3)
Supplement: Supplementary file 5 — Reporting Summary [file 41467_2024_49666_MOESM5_ESM.pdf]

nature portfolio

Corresponding author(s): Liang Kou

Last updated by author(s): Jun 8, 2024

Reporting Summary

Nature Portfolio wishes to improve the reproducibility of the work that we publish. This form provides structure for consistency and transparency in reporting. For further information on Nature Portfolio policies, see our Editorial Policies and the Editorial Policy Checklist.

Statistics

For all statistical analyses, confirm that the following items are present in the figure legend, table legend, main text, or Methods section.

n/a	Confirmed	
☐	☒	The exact sample size (*n*) for each experimental group/condition, given as a discrete number and unit of measurement
☐	☒	A statement on whether measurements were taken from distinct samples or whether the same sample was measured repeatedly
☐	☒	The statistical test(s) used AND whether they are one- or two-sided<br>*Only common tests should be described solely by name; describe more complex techniques in the Methods section.*
☐	☒	A description of all covariates tested
☐	☒	A description of any assumptions or corrections, such as tests of normality and adjustment for multiple comparisons
☐	☒	A full description of the statistical parameters including central tendency (e.g. means) or other basic estimates (e.g. regression coefficient) AND variation (e.g. standard deviation) or associated estimates of uncertainty (e.g. confidence intervals)
☐	☒	For null hypothesis testing, the test statistic (e.g. *F*, *t*, *r*) with confidence intervals, effect sizes, degrees of freedom and *P* value noted<br>*Give P values as exact values whenever suitable.*
☒	☐	For Bayesian analysis, information on the choice of priors and Markov chain Monte Carlo settings
☒	☐	For hierarchical and complex designs, identification of the appropriate level for tests and full reporting of outcomes
☒	☐	Estimates of effect sizes (e.g. Cohen's *d*, Pearson's *r*), indicating how they were calculated

Our web collection on statistics for biologists contains articles on many of the points above.

Software and code

Policy information about availability of computer code

Data collection	Root samples were scanned using an Epson Expression 10000 XL scanner (Seiko Epson Corporation, Suwa, Nagano, Japan).<br>Root traits were measured using WinRHIZO Arabidopsis version 2012b (Regents Instruments Inc., Quebec, Canada).<br>Root N concentration was measured using a Vario Macro cube elemental analyser (Elementar Analysensysteme GmbH, Langenselbold, Germany). Root anatomical traits were photographed with a compound microscope(DM2500, DFC450, Leica, Weztlar, Germany) and measured using ImageJ (NIH Image, Bethesda, MD, USA).
Data analysis	Data analysis were performed using the R software(v.4.1.3; https://www.R-project.org/). Details were reported in the Method section. Briefly, we used open-source R packages for statistical analyses, such as phytools, V. PhyloMaker, ape, stats,picante, caper, ggplot2, etc.

For manuscripts utilizing custom algorithms or software that are central to the research but not yet described in published literature, software must be made available to editors and reviewers. We strongly encourage code deposition in a community repository (e.g. GitHub). See the Nature Portfolio guidelines for submitting code & software for further information.

Data

Policy information about availability of data

All manuscripts must include a data availability statement. This statement should provide the following information, where applicable:

- Accession codes, unique identifiers, or web links for publicly available datasets
- A description of any restrictions on data availability
- For clinical datasets or third party data, please ensure that the statement adheres to our policy

Data and R code in the support of these findings are available via the Dryad Digital Repository (https://doi.org/doi:10.5061/dryad.p2ngf1vzn)

Research involving human participants, their data, or biological material

Policy information about studies with human participants or human data. See also policy information about sex, gender (identity/presentation), and sexual orientation and race, ethnicity and racism.

Reporting on sex and gender	N/A
Reporting on race, ethnicity, or other socially relevant groupings	N/A
Population characteristics	N/A
Recruitment	N/A
Ethics oversight	N/A

Note that full information on the approval of the study protocol must also be provided in the manuscript.

Field-specific reporting

Please select the one below that is the best fit for your research. If you are not sure, read the appropriate sections before making your selection.

☐ Life sciences ☐ Behavioural & social sciences ☒ Ecological, evolutionary & environmental sciences

For a reference copy of the document with all sections, see nature.com/documents/nr-reporting-summary-flat.pdf

Ecological, evolutionary & environmental sciences study design

All studies must disclose on these points even when the disclosure is negative.

Study description	To reveal the strategies for belowground resource acquisition, we integrated root morphological, architectural, anatomical and chemical traits, and decomposition rate. We sampled absorptive roots of 90 angiosperm tree species spanning two temperate and two subtropical forests in China. We aimed to test root trait- relationship at the species level.
Research sample	In total, 270 individuals of 90 tree species were sampled; 270 microcosms (90 tree species × 3 replicates) were set up in this study. Similar sample sizes have been adequate in past research within the same field. We used the mean values of root traits for each species in subsequent analyses, because we focus on the relationship among different root traits at the level of plant species. All procedures are standard.
Sampling strategy	For each species, three well-developed trees with a similar diameter at breast height were randomly selected, with a spacing of at least 10 m apart to ensure independence; thus, 270 individuals of 90 tree species were sampled. Tree species selected at each site were the dominant species and the most important in terms of the composition of the local plant communities. The surface mineral soil (0-20 cm) in the sampling area was loosened carefully using a pickaxe and spade, and then the intact fine roots (including at least the first five root orders) were excavated by exposing the main lateral roots, traced back to individual trees and cleaned following the approach of precious study.
Data collection	Data collection was performed by Jiajia Zheng, Han Yan, Lei Jiang, Ning Ma during the field and laboratory experiment.<br>Briefly, Jiajia Zheng, Han Yan and Lei Jiang used instruments for root trait measurements, and Ning Ma used paper and computer for recording. Jiajia Zheng set up the indoor microcosm experiment with the help of Lei jiang, and Han Yan and Ning Ma weighed and recorded them on a balance.
Timing and spatial scale	We conducted field sampling from August to October 2018 and 2020, the peak period of vegetation growth.<br>We conducted our study across four sites, including Mengluan Forestry Station (41°44′N, 117°05′E) in Hebei Province; (ii) Taibai Mountain (33°49′-34°10′N, 107°19′-107°58′E) in Shanxi Province; (iii) Qianyanzhou Ecological Station (26°44′N, 115°03′E) in Jiangxi Province; and (iv) Yangming Mountain (25°39′N, 114°18′E) in Jiangxi Province, which belong to temperate or subtropical forest ecosystems, the elevation of these sites ranges from 102 to 2100 m.

Data exclusions	Once we compiled the dataset according to our criteria, we did not deliberately delete any data.
Reproducibility	We used comprehensive approach that involving the standardized sampling procedures and experimental design, independent replications of root sampling, rigorous statistical analysis. The majority of our replication attempts were successful. R codes and root traits data have been deposited in the Dryad database and can be accessed at https://doi.org/10.5061/dryad.p2ngf1vzn.
Randomization	For each species, three well-developed trees with a similar diameter at breast height were randomly selected with a spacing of at least 10 m apart to ensure independence. In the root decomposition experiment, each litter bag was randomly embedded in the corresponding microcosm.
Blinding	Blinding was not applicable since this is a field investigation experiment

Did the study involve field work? ☒ Yes ☐ No

Field work, collection and transport

Field conditions	The study was conducted in temperate (two sites) and subtropical (two sites) forests in China, with a mean annual temperature of 2.0–17.9 ℃ and a mean annual precipitation of 470-1587mm.
Location	These four sites were located at (i) Mengluan Forestry Station (41°44′N, 117°05′E) in Hebei Province; (ii) Taibai Mountain (33°49′-34°10′N, 107°19′-107°58′E) in Shanxi Province; (iii) Qianyanzhou Ecological Station (26°44′N, 115°03′E) in Jiangxi Province; and (iv) Yangming Mountain (25°39′N, 114°18′E) in Jiangxi Province. The elevation of these sites ranges from 102 to 2100 m.
Access & import/export	This research complies with all relevant ethical regulations. All experimentations and soil and root sampling have been approved by the local authorities.
Disturbance	Disturbance was limited by backfilling the topsoil after sampling under the supervision of the local forest managers.

Reporting for specific materials, systems and methods

We require information from authors about some types of materials, experimental systems and methods used in many studies. Here, indicate whether each material, system or method listed is relevant to your study. If you are not sure if a list item applies to your research, read the appropriate section before selecting a response.

Materials & experimental systems

n/a	Involved in the study
☒	☐ Antibodies
☒	☐ Eukaryotic cell lines
☒	☐ Palaeontology and archaeology
☒	☐ Animals and other organisms
☒	☐ Clinical data
☒	☐ Dual use research of concern
☒	☐ Plants

Methods

n/a	Involved in the study
☒	☐ ChIP-seq
☒	☐ Flow cytometry
☒	☐ MRI-based neuroimaging

Plants

Seed stocks	For each species, three well-developed trees with a similar diameter at breast height were randomly selected, with a spacing of at least 10 m apart to ensure independence, and the intact fine roots were excavated by exposing the main lateral roots, traced back to individual trees and cleaned .
Novel plant genotypes	N/A.
Authentication	N/A.
